# Multiplex immunoassay characterization and species comparison of inflammation in acute and non-acute ischemic infarcts in human and mouse brain tissue

**DOI:** 10.1186/s40478-016-0371-y

**Published:** 2016-09-06

**Authors:** Thuy-Vi V. Nguyen, Jennifer B. Frye, Jacob C. Zbesko, Kristina Stepanovic, Megan Hayes, Alex Urzua, Geidy Serrano, Thomas G. Beach, Kristian P. Doyle

**Affiliations:** 1 Department of Immunobiology, University of Arizona College of Medicine, Tucson, AZ USA; 2 Department of Neurology, University of Arizona College of Medicine, Tucson, AZ USA; 3 Arizona Center on Aging, University of Arizona College of Medicine, Tucson, AZ USA; 4 Banner Sun Health Research Institute, Sun City, AZ USA

**Keywords:** Inflammation, Stroke, Liquefactive necrosis, Chronic infarcts, Cytokines

## Abstract

This study provides a parallel characterization of the cytokine and chemokine response to stroke in the human and mouse brain at different stages of infarct resolution. The study goal was to address the hypothesis that chronic inflammation may contribute to stroke-related dementia. We used C57BL/6 and BALB/c mice to control for strain related differences in the mouse immune response. Our data indicate that in both mouse strains, and humans, there is increased granulocyte macrophage colony-stimulating factor (GM-CSF), interleukin-6 (IL-6), interleukin-12 p70 (IL-12p70), interferon gamma-induced protein-10 (IP-10), keratinocyte chemoattractant/interleukin-8 (KC/IL-8), monocyte chemoattractant protein-1 (MCP-1), macrophage inflammatory protein-1α (MIP-1α), macrophage inflammatory protein-1β (MIP-1β), regulated on activation, normal T cell expressed and secreted (RANTES), and Tumor necrosis factor-α (TNF-α) in the infarct core during the acute time period. Nevertheless, correlation and two-way ANOVA analyses reveal that despite this substantial overlap between species, there are still significant differences, particularly in the regulation of granulocyte colony-stimulating factor (G-CSF), which is increased in mice but not in humans. In the weeks after stroke, during the stage of liquefactive necrosis, there is significant resolution of the inflammatory response to stroke within the infarct. However, CD68+ macrophages remain present, and levels of IL-6 and MCP-1 remain chronically elevated in infarcts from both mice and humans. Furthermore, there is a chronic T cell response within the infarct in both species. This response is differentially polarized towards a T helper 1 (Th1) response in C57BL/6 mice, and a T helper 2 (Th2) response in BALB/c mice, suggesting that the chronic inflammatory response to stroke may follow a different trajectory in different patients. To control for the fact that the average age of the patients used in this study was 80 years, they were of both sexes, and many had suffered from multiple strokes, we also present findings that reveal how the chronic inflammatory response to stroke is impacted by age, sex, and multiple strokes in mice. Our data indicate that the chronic cytokine and chemokine response to stroke is not substantially altered in 18-month old compared to 3-month old C57BL/6 mice, although T cell infiltration is attenuated. We found a significant correlation in the chronic cytokine response to stroke in males and females. However, the chronic cytokine response to stroke was mildly exacerbated by a recurrent stroke in both C57BL/6 and BALB/c mice.

## Introduction

Stroke is the fifth leading cause of death in the United States, although many patients survive. About 8 % of those who survive go on to experience another stroke within a year [32]. Of the seven million Americans currently living with the after effects of stroke, 30 % suffer from delayed cognitive dysfunction, or dementia, that worsens progressively after the stroke [1, 5, 29]. Patients who develop dementia after stroke exhibit a leaky blood-brain-barrier (BBB) and signs of increased inflammation in their blood [22, 27, 43]. Those findings support the hypothesis that a chronic inflammatory response to necrotic brain tissue might be a cause of delayed cognitive decline. However, a major challenge to testing this hypothesis is that in both animal models and humans, neither the resolution nor the characteristics of late inflammation after cerebral infarction are well defined [17, 25, 26, 30, 31]. In rodents, most research to date has focused on inflammation within the first few weeks after stroke, the length of time it takes for the core of the injury to be sealed by glial scarring [25, 26]. In humans, there has been far less research and the long-term sequential pathology of inflammation in the brain after ischemic stroke has only been described by histology [31]. Although histology has revealed that there is persistent immune cell infiltration, sometimes decades after stroke, in approximately 40 % of human cerebral infarcts, the identity of the immune cells and what cytokines and chemokines they may be producing is unknown [31].

It was recently demonstrated that there is a chronic immune response to stroke that lasts for at least 7 weeks in mice, and that targeting at least one component of this response, the B cell component, can prevent the development of delayed cognitive deficits [9]. As a follow-up to that study, and to further address the hypothesis that one of the mechanisms that contributes to delayed cognitive dysfunction after stroke is a chronic inflammatory response that persists at the site of the lesion, in turn causing bystander damage to surrounding tissue, we present here a characterization of the cytokine and chemokine response to stroke in the human brain at different stages of infarction. To bridge this study with preclinical stroke research we also characterized, in parallel, the cytokine and chemokine response to stroke in the mouse brain at different stages of infarction. We used mice since most basic and preclinical stroke research is conducted in mouse models of stroke. Furthermore, because there are significant differences in the inflammatory response to disease and injury in different mouse strains, we used two popular strains, C57BL/6 and BALB/c, to control for mouse strain-related heterogeneity in the inflammatory response to stroke [4].

Nearly 75 % of all strokes occur in patients over the age of 65 years, and approximately 25 % of the nearly 800,000 strokes experienced in the United States every year are recurrent strokes [32]. Accordingly, the human post-mortem tissue used in this study came from elderly men and women, some of whom suffered from multiple strokes. To control for this heterogeneity, and serve as an additional bridge between clinical and preclinical stroke research, we also present data that reveals how the chronic inflammatory response to stroke is impacted by age and sex in C57BL/6 mice, and by multiple strokes in both C57BL/6 and BALB/c mice. Sieber and collegues previously showed that increases of TNFα, IL-1β, MIP-1α, MCP-1, and IL-6 are attenuated 7 days following stroke in aged C57BL/6 mice, and that older mice have smaller infarct volumes [42]. Other studies have shown that the mechanisms of damage following stroke are sexually dimorphic (reviewed in [23, 37]). However, no studies have yet reported how the chronic cytokine and chemokine response to stroke is altered in any animal models of recurrent stroke.

Our findings reveal that there is substantial overlap in the acute cytokine and chemokine response to stroke in mice and humans, albeit there are key differences. For example, in humans compared to mice, G-CSF and IL-6 appear differentially regulated. They also reveal that there is substantial overlap in the chronic inflammatory response to stroke, which our data suggest resolves more slowly in the brain than in other tissues in both humans and mice. However, there are still key differences, for example, C57BL/6 mice develop a chronic Th1 polarized T cell response, whereas BALB/c mice develop a chronic Th2 polarized T cell response, and humans appear to be more heterogeneous in their chronic adaptive immune response to stroke than either strain.

Our findings also show that the inflammatory response to stroke is remarkably consistent in aged mice, although fewer T cells infiltrate the brain in aged animals, that it significantly correlates in males and females, and that the inflammatory response to a recurrent stroke is mildly exacerbated in C57BL/6 and BALB/c mice. Together, these data provide a resource for target validation for preclinical stroke studies by providing a parallel map of the cytokine and chemokine response to stroke during different stages of infarct resolution in humans, as well two commonly used mouse strains, in the presence and absence of two common stroke co-morbidities.

## Material and methods

### Human tissue

**Fig. 1:**
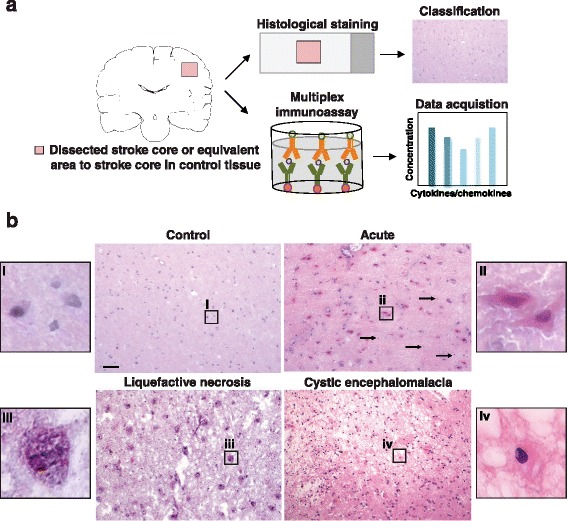
Staging of human brain tissue. **a** Infarcts and equivalent areas of brain from controls, were dissected and cut into two pieces. Each piece was allocated for histological H&E staining and staging, or multiplex immunoassay. **b** Representative images of staging criteria. (*i*) Normal control tissue, (*ii*) acute infarct with eosinophilic neurons clearly distinguishable. Some neuronal nuclei also display evidence of pyknosis and karyorrhexis (*arrows*). (*iii*) Lesion at the stage of liquefactive necrosis, demonstrated by the presence of foamy macrophages. (*iv*) Lesion at the stage of cystic encephalomalacia, evidenced by the presence of reactive astrocytes (an example is shown in the *box*) and a network of glial fibers. Scale bar, 50 μm

Acute and chronic stroke infarcts dissected from fresh frozen brain tissue (5 g blocks), along with location-matched tissue from age-matched controls, were obtained from the Banner Sun Health Research Institute (Sun City, AZ) [3], the University of California at Los Angeles Human Brain and Spinal Fluid Resource Center (Veterans Affairs, Greater Los Angeles Healthcare System, Los Angeles, CA), and the University of Miami Brain Endowment Bank (Miller School of Medicine, Miami, FL). Tissue samples came from 55 post-mortem brains; 33 samples came from patients with a clinical and neuropathological diagnosis of either acute or chronic stroke, and 23 samples from age-matched control patients. Samples came from brain regions mainly comprising the frontal cortex and hippocampus, with others from the temporal cortex, striatum, and internal capsule. All patients with Alzheimer’s disease, Parkinson’s disease, Huntington’s disease, multiple sclerosis, or any other confounding neurological disease were excluded. Both male and female patients were included. The mean age at death was 79.9 years, and the mean autolysis time was 13.2 h (see Table 1 for patient information). The autolysis cut-off time for this study was 24 h. Research was conducted in compliance with policies and principles contained in the Federal Policy for the Protection of Human Subjects. To determine if the dissected lesions were at the stage of acute injury, liquefactive necrosis, or cystic encephalomalacia, tissue samples were staged by cross-checking clinical information with histological assessment by hematoxylin and eosin (H&E) staining using standard techniques. Tissue samples then underwent processing for immunostaining and multiplex immunoassays (Fig. 1a).

**Table 1:** Patient information

*N* = 55 patients	Normal control patients	Acute stroke patients	Liquefactive necrosis patients	Cystic encephalomalacia patients
Age in years (range)	77.4 (53–92)	76.2 (55–91)	86.8 (77–106)^a^	79.8 (62–92)
N (females/males)	23 (8/15)	5 (2/3)	18 (9/9)	9 (7/2)
Autolysis time in hours (range)	11.7 (2.8–24)	9.6 (3–23.9)	5.3 (2.3–9.5)^b^	6.8 (2.3–19.8)

^a^ *p* ≤ 0.05 and ^b^ *p* ≤ 0.01 versus normal control patients as analyzed by a t-test and corrected for multiple comparisons

### Staging criteria

Lesions were classified as acute if there was evidence of eosinophilic neurons, and if neuronal nuclei displayed evidence of pyknosis and karyorrhexis. The classification of acute was also confirmed by the presence of swollen, faded glia and endothelial cells, and evidence of myelin fiber disintegration. Lesions at the stage of liquefactive necrosis were classified by the presence of foamy macrophages, and if around the periphery of the lesion, astrocytes had multiplied and developed interconnected extensions to form the astroglial scar. Lesions were classified as at the stage of cystic encephalomalacia if there was evidence that the lesion had been predominantly resorbed leaving a cavity surrounded by a dense network of glial fibers and blood vessels (Fig. 1b).

### Mice

Adult male C57BL/6 mice and adult male BALB/c mice (10–12 weeks old) were purchased from The Jackson Laboratory (Bar Harbor, ME), with the exception of one cohort of male 18-month old C57BL/6 mice that were obtained from the National Institute on Aging (Bethesda, MD), and one cohort of female ﻿C57BL/6 mice﻿ (10-12 weeks old) that were obtained from Harlan/Envigo (Indianapolis, IN). All animals were housed under a 12-h light/dark schedule with *ad libitum* access to food and water. All animal experiments were designed to ensure humane endpoints and minimize numbers of mice and suffering. Allocation of mice into stroke and sham experimental groups was performed randomly. Mice underwent stroke surgery at 12 weeks or 18 months of age. For mice that underwent two stroke surgeries, there was a recovery period of 3 weeks before the second stroke surgery was performed on the side contralateral to the first stroke. Mice were then euthanized at 24 h, 1 week, or 7 weeks following stroke. For euthanasia, mice were intraperitoneally injected with a mixture of 100 mg/kg ketamine, 20 mg/kg xylazine, and 3 mg/kg acepromazine, and intracardinally perfused with 0.9 % saline. Brains were extracted, and the infarct was dissected for flow cytometry, or snap frozen in liquid nitrogen for multiplex immunoassays. Alternatively, whole brains were post-fixed in 4 % paraformaldehyde for immunostaining.

### Stroke surgery

In BALB/c mice, distal middle cerebral artery occlusion (DMCAO) was performed, as previously described [8]. Sham surgeries were identical to stroke surgeries, except for ligation of the DMCA. C57BL/6 mice were given 9 % oxygen for 45 min immediately after DMCAO. We refer to this variation of DMCAO as DH stroke (DMCAO + hypoxia). This period of hypoxia induces vasoconstriction and compensates for extensive collateralization between the anterior cerebral artery and MCA present in C57BL/6 mice, and makes stroke size in C57BL/6 mice equivalent to stroke size in BALB/c mice following DMCAO alone [8]. For all experiments mice were subcutaneously injected with 0.1 mg/kg buprenorphine before surgery, anesthesized with 1–3 % isoflurane inhalation during surgery, and injected with 25 mg/kg cefazolin and 1 mg/kg buprenorphine SR immediately after surgery. Mice that underwent sham DH stroke were also given 9 % oxygen for 45 min immediately after surgery. Core body temperature was maintained at 37 °C throughout surgery and hypoxia in C57BL/6 mice.

### Human immunohistochemistry

Human brain sections were immunostained using standard techniques using an anti-CD20 (1:500; Dako, Cirpinteria, CA), anti-CD3 (1:500; Dako), anti-CD4 (1:500; Ventana Medical Systems, Tucson, AZ), anti-CD68 (1:500; Dako), and anti-CD8 (1:500; Ventana Medical Systems, Tucson, AZ) antibodies. Total CD20+, CD3+, CD4+, and CD8+ cells present in each slide (one section per patient, *N* = 7 patients with liquefactive necrosis strokes) were counted using a Motic BA410 light microscope coupled with a MoticamPro 282A camera (Motic Images Plus 2.0 software) and a 10× objective, and divided by the area of tissue sample present on the slide to obtain the number of positivity stained cells per cm^2^.

### Mouse immunohistochemistry

Frozen coronal sections (40 μm) were taken through the entire brain using a Microm HM 450 sliding microtome (Thermo Fisher Scientific, Walthman, MA). Primary antibodies included anti-B220 (biotinylated, 1:500; BD Biosciences, Franklin Lakes, NJ), anti-CD68 (1:1000; AbD Serotec, Raleigh, NC), and anti-CD3e (the ε chain of the T cell receptor associated CD3 complex, 1:500; BD Biosciences). Briefly, free floating sections were immunolabeled with antibodies in conjuction with ABC Vector Elite and 3,3’-diaminobenzidine kits for visualization. B220+ and CD3e + cells were counted using the NIH Image J (Media Cybernetics, Rockville, MD) cell count function in the stroke core in 2 sections (bregma −0.46 mm and −1.94 mm) per mouse, and 3 non-overlapping fields (112 μm × 85 μm using a 20× objective). Due to the denseness and intensity of CD68 immunoreactivity within each lesion, CD68 was quantified by measuring the percent area occupied by CD68 (*n* = 5–8 mice per group for CD3e-immunostaining, *n* = 4–12 mice per group for B220-immunostaining, and *n* = 4–7 mice per group for CD68-immunostaining).

### Flow cytometry

Stroke lesions from brains harvested 7 weeks following stroke were dissected and combined for each mouse strain (*n* = 9 lesions pooled from C57BL/6 mice, and *n* = 6 lesions pooled from BALB/c mice) to ensure sufficient number of cells. To isolate immune cells, the pooled lesions were incubated in 3 % FBS/DMEM, followed by dissociation of cells in ACK lysing buffer (Thermo Fisher Scientific). Re-suspended cells were treated with 1 μM ionomycin (Thermo Fisher Scientific), 50 ng/ml phorbol 12-myristate 13-acetate (Thermo Fisher Scientific), and 1 μl/ml GolgiPlug (BD Biosciences) for 4 h. Cells were then re-suspended in Fixation/Permeabilization Buffer (R&D Systems, Minneapolis, MN), after which 10 μl of each antibody (except for 1 μl each of IL-17-Alexa Fluor 700, T-bet-PE, CD4-Pacific Blue, and FoxP3-Alexa Fluor 700), or corresponding isotype control antibody, was added. Antibodies included: IFN-γ-CFS, (R&D Systems, Minneapolis, MN), IL-12 Rβ2-APC (R&D Systems), T-bet-PE (eBioscience, San Diego, CA), CD4-Pacific Blue (BioLegend, San Diego, CA), IL-17-Alexa Fluor 700 (BioLegend, San Diego, CA), IL-4 R-CFS (R&D Systems), STAT6-APC, (R&D Systems), IL-5-PE (R&D Systems), CD4-Pacific Blue, and FoxP3-Alexa Fluor 700, (R&D Systems). Cells were incubated for 45 min. They were then analyzed using a FACScan flow cytometer (BD Biosciences), and quantified using FlowJo software (Tree Star, Ashland, OR). Gating strategy: Cells were gated by forward scatter and side scatter to identify lymphocytes, using lymphocytes isolated from the spleen as a positive control. They were then gated on CD4+ expression.

### Multiplex immunoassay

Frozen tissue from each human (*n* = 5–23 patients per group; see Table 1 for exact n size per group) and mouse (*n* = 5–10 mice per group; see Table 2 for exact n size per group) cohort were homogenized in ice-cold phosphate buffered saline lysis solution containing 1 % triton-X and 0.1 % sodium deoxycholate, complete with Protease Inhibitor Cocktail (Sigma-Aldrich, Saint Louis, MO) and Phosphatase Inhibitor Cocktail 2 (Sigma-Aldrich) at a ratio of 1:100. Following centrifugation, the total protein concentration of the supernatant was measured using a Direct Detect Infrared Spectrometer. Cytokines and chemokines were then detected and quantified by multiplex immunoassays. Human high sensitivity and mouse multiplex magnetic bead kits were purchased from EMD Millipore (Billerica, MA) and used according to the manufacturer’s recommendations. Each lysate sample, standard, and quality control was measured in duplicate. Plates were read using a MAGPIX instrument (Luminex, Austin, TX), and results were analyzed using MILLIPLEX Analyst 5.1 software (EMD Millipore). For those analytes that were below the lower limit of detection, the lower limit of detection values were used for data analysis. Concentrations of cytokines and chemokines were normalized to concentrations of total protein in lysates.

**Table 2:** Number of mice used for cytokine and chemokine profile data

C57BL/6 mice (*n* = 90)				BALB/c mice (*n* = 62)			
Time-point	Experimental group	Age (months)	n	Time-point	Experimental group	Age (months)	n
24 h	Sham male mice	3	6	24 h	Sham male mice	3	6
		18	--			18	--
	1 Stroke male mice	3	6		1 Stroke male mice	3	8
		18				18	--
	2 Strokes male mice	3	6		2 Strokes male mice	3	9
		18	--			18	--
Week 1	Sham male mice	3	6	Week 1	Sham male mice	3	5
		18	--			18	--
	1 Stroke male mice	3	8		1 Stroke male mice	3	6
		18	--			18	--
	2 Strokes male mice	3	7		2 Strokes male mice	3	8
		18	--			18	--
Week 7	Sham male mice	3	10	Week 7	Sham male mice	3	6
		18	5			18	--
	1 Stroke male mice	3	12		1 Stroke male mice	3	7
		18	8			18	--
	2 Strokes male mice	3	5		2 Strokes male mice	3	7
		18	--			18	--
	Sham female mice	3	5				
		18	--				
	1 Stroke female mice	3	6				

Total number of mice, *n* = 152

### Statistical analysis

**Fig. 2:**
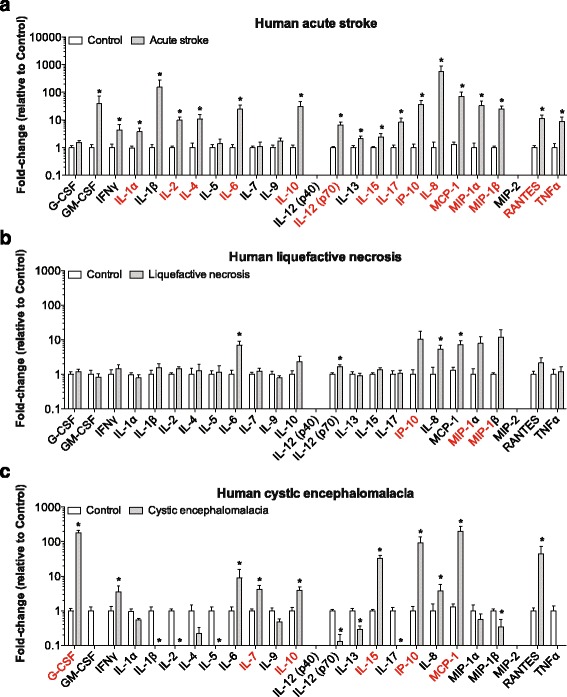
Cytokine and chemokine levels in human infarcts at the stages of acute stroke, liquefactive necrosis, and cystic encephalomalacia. Graphs show the levels of 25 cytokines and chemokines in **a** acute infarcts (*n* = 5), **b** infarcts at the stage of liquefactive necrosis (*n* = 18), and **c** infarcts at the stage of cystic encephalomalacia (*n* = 9). Data is presented as a fold-change compared to equivalent brain regions from control patients. Data represent mean ± SEM. *p < 0.05 versus controls by Student’s t-test when uncorrected for multiple comparisons. *Red* indicates cytokines and chemokines that were significantly different when corrected for multiple comparisons

**Fig. 3:**
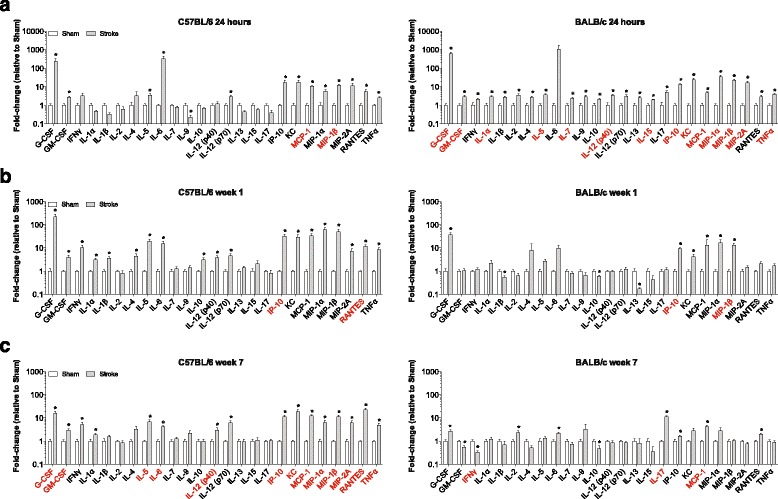
Cytokine and chemokine levels in C57BL/6 and BALB/c mouse infarcts at the stages of acute stroke (24 h), sub-acute stroke (1 week), and liquefactive necrosis (7 weeks). Graphs show the levels of 25 cytokines and chemokines in **a** acute infarcts, **b** sub-acute infarcts, and **c** infarcts at the stage of liquefactive necrosis (n’s provided in Table 2). Data is presented as a fold-change compared to equivalent cortex dissected from sham mice from each strain. Data represent mean ± SEM. *p < 0.05 versus sham by Student’s t-test when uncorrected for multiple comparisons. *Red* indicates cytokines and chemokines that are significantly different when corrected for multiple comparisons

**Fig. 4:**
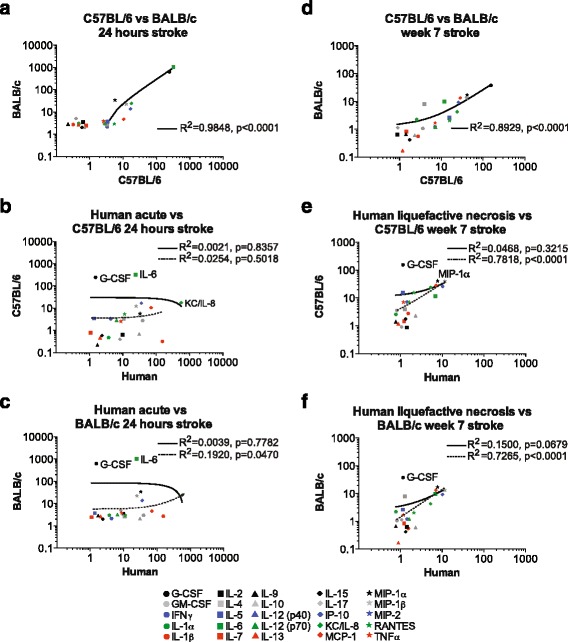
Correlation of cytokine and chemokine levels in mouse and human acute infarcts, and infarcts at the stage of liquefactive necrosis. Data from Figs. 2 and 3 have been replotted to provide a direct comparison of cytokine and chemokine levels in acute mouse and human infarcts (**a**, **b**, and **c**), and human and mouse infarcts at the stage of liquefactive necrosis (**d**, **e**, and **f**). The specific cytokines and chemokines that are significantly different between each strain, and different between humans and each strain are provided in Table 4. The solid lines depict the correlation when all 25 cytokines and chemokines are taken into consideration for the calculation of the Pearson’s R values. The *dotted lines* depict the correlation when the cytokines and chemokines that are significantly different between species by a two-way ANOVA corrected for multiple comparisons are excluded from the calculation of the Pearson’s R values

**Fig. 5:**
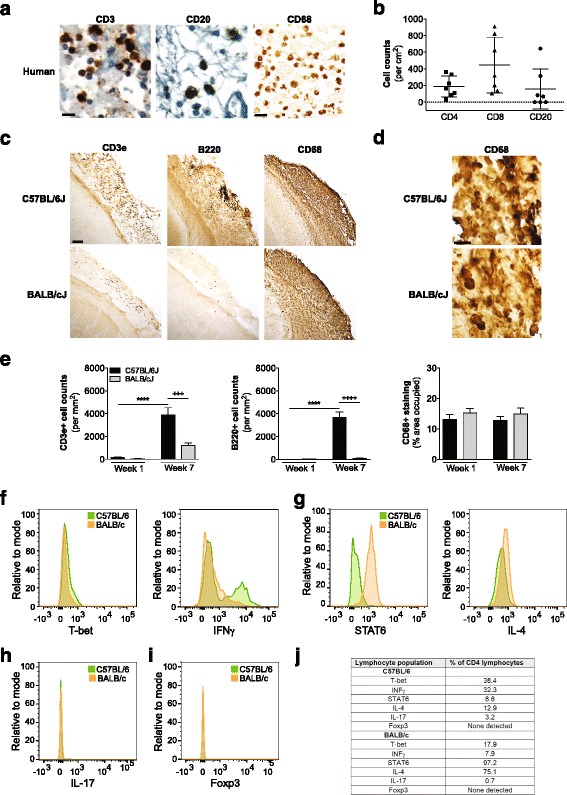
Immune cell composition in infarcts at the stage of liquefactive necrosis in humans and mice. **a** Representative images of CD3+ T-lymphocyte, CD20+ B-lymphocyte, and CD68+ macrophage/microglia infiltration in human infarcts at the stage of liquefactive necrosis. Scale bars, 10 μm (human, CD3 and CD20 images) and 30 μm (human, CD68 image). **b** Quantification of CD4+ and CD8+ T-lymphocyte, and CD20+ B-lymphocyte infiltration into the infarcts. **c** Representative images of CD3+ T-lymphocytes, B220+ B-lymphocytes, and CD68+ macrophages/microglia in the infarcts of C57BL/6 and BALB/c mice at 7 weeks post-stroke. Scale bar,100 μm. **d** Higher magnification images of CD68+ macrophages/microglia in the infarct to reveal individual cells. Scale bar, 25 μm. **e** Quantification of CD3+ T-lymphocyte infiltration, B220+ B-lymphocyte infiltration, and CD68 immunostaining in mouse infarcts. ****p < 0.0001 compared to week 1, +++ *p* < 0.001 compared to BALB/c, ++++ *p* < 0.0001 compared to BALB/c. **f** Flow cytometry on cells gated by CD4, using markers of Th1 cells (T-bet and IFNγ), demonstrates that at 7 weeks post-stroke, the CD4+ T cell response in the infarct in C57BL/6 mice is more polarized towards a Th1 response than in BALB/c mice. **g** Flow cytometry on cells gated by CD4, using markers of Th2 cells (STAT6 and IL-4), demonstrates that at 7 weeks post-stroke, the T cell response in the infarct in BALB/c mice is more polarized towards a Th2 response than in C57BL/6 mice. **h** There were few CD4+ IL-17+ cells detected in the infarct of both strains. **i** There were no CD4+ Foxp3 cells detected in the infarct in either strain. **j** Table showing the % CD4 lymphocytes positive for each marker

**Fig. 6:**
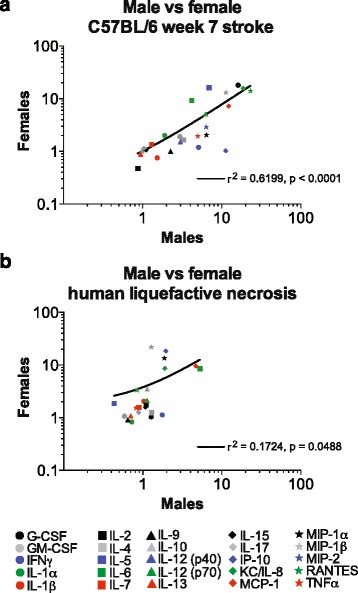
Impact of sex on the chronic inflammatory response to stroke in C57BL/6 mice and humans. **a** Comparison of cytokine and chemokine levels in infarcts at the stage of liquefactive necrosis from male and female C57BL/6 mice reveals that there is a significant correlation between each sex. **b** Comparison of cytokine and chemokine levels in infarcts at the stage of liquefactive necrosis from male and female stroke patients reveals that there is also a significant correlation between each sex in humans

**Fig. 7:**
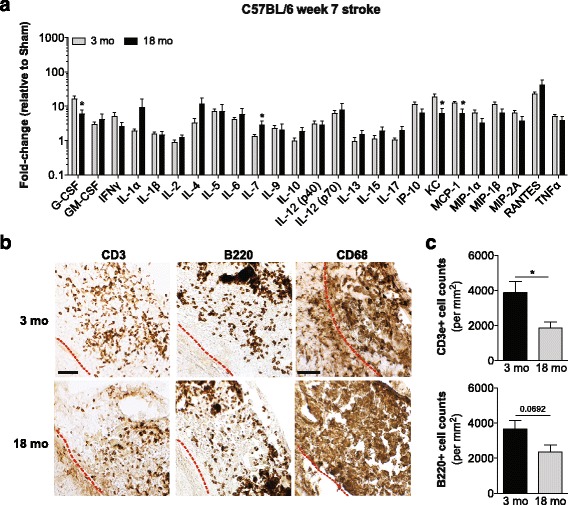
Impact of age on the chronic inflammatory response to stroke in C57BL/6 mice. **a** Comparison of the levels of 25 cytokines and chemokines in infarcts at the stage of liquefactive necrosis dissected from 3-month old and 18-month old C57BL/6 mice at 7 weeks post-stroke. Data are expressed as a fold-change relative to age matched sham control values. Data represent mean ± SEM. There is no significant difference in the overall cytokine and chemokine profile at the stage of liquefactive necrosis between 3-month old and 18-month old mice by two-way ANOVA. Cytokines and chemokines that are significantly different between the 3-month old and 18-month old mice corrected for multiple comparisons are denoted by an asterisk (*p < 0.05 versus 3-month old mice). **b** Representative images of CD3+ T-lymphocytes, B220+ B-lymphocytes, and CD68+ macrophages/microglia in the infarcts of 3-month old and 18-month old C57BL/6 mice at 7 weeks post-stroke. Scale bar, 50 μm. *Red dotted lines* indicate locations of the glial scar in each image. **c** Quantification of CD3+ T-lymphocyte and B220+ B-lymphocyte infiltration into the mouse infarcts

**Fig. 8:**
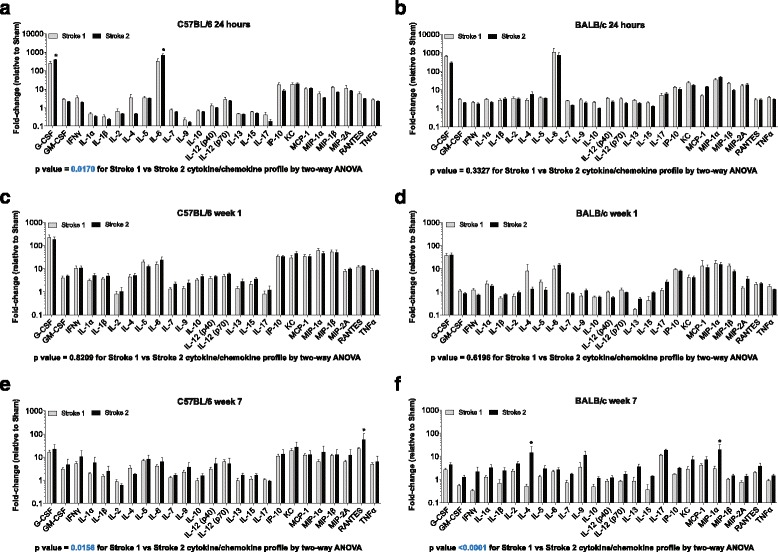
Impact of recurrent stroke on the acute, sub-acute and chronic inflammatory response to stroke in C57BL/6 and BALB/c mice. Graphs show a comparison of the levels of 25 cytokines and chemokines in acute infarcts (**a** and **b**), sub-acute infarcts (**c** and **d**), and infarcts at the stage of liquefactive necrosis (**e** and **f**) dissected from a first or second stroke. In mice that underwent two stroke procedures, each stroke was separated by 3 weeks. Only the infarct at the site of the second stroke was compared to the infarct from mice that had undergone a single stroke procedure. Data are expressed as a fold-change relative to values from mice that underwent a single sham surgery. *Blue text* denotes where there is a significant difference in the overall cytokine and chemokine profile between the first and second stroke by two-way ANOVA. Cytokines and chemokines that are significantly different between the first and second stroke, corrected for multiple comparisons, are denoted by an *asterisk*

For immunohistochemistry, blinding was not possible because of the stroke lesion being clearly visible in stroke patients versus normal control patients, as well as stroke versus sham mouse groups. However, multiplex immunoassay analyses were performed with blinding to the stage of human strokes, as well as the ages, strains, time-points, and one versus two strokes in mice. Data are expressed as mean ± SEM. Statistical analyses were performed with Prism 6.0 software (GraphPad, San Diego, CA), with the level of significance set at *p* < 0.05. Mouse immunohistochemistry data were analyzed using a two-way ANOVA, followed by *post-hoc* Tukey’s multiple comparison testing (Fig. 5e), ﻿or tw﻿o-tailed unpaired t-test (Fig. 7c). Differences in cytokine and chemokine concentrations between experimental groups (e.g., normal control versus acute stroke patients, or sham versus stroke mice) were analyzed by Student’s t-test both with and without correction for multiple comparisons using the Holm-Sidak method. Both the uncorrected and corrected statistics are provided in order to impart additional information with regard to Type I error from this dataset. Due to the multiple hypotheses tests, the uncorrected data may include differences that are false positives. The corrected data may exclude legitimate differences because of the small sample size. The rationale for providing the uncorrected data is to facilitate cross-referencing the human and mouse data for future studies. Differences that were significant without correction for multiple comparisons are denoted in the graphs by asterisks. Differences that were significant with correction for multiple comparisons are referenced in the Results section and denoted with red text in the graphs (Figs. 2 and 3). To test for significant correlation in overall cytokine and chemokine profiles between experimental groups that include all 25 analytes (e.g., C57BL/6 mice at the 24 h time-point versus BALB/c mice at the 24 h time-point, or human acute stroke patients versus C57BL/6 mice at the 24 h time-point), R squared and P values were calculated using Prism 6.0 (Figs. 4 and 6). To look for significant differences in individual cytokines and chemokines between experimental groups that failed to correlate, datasets were analyzed by two-way ANOVA corrected for multiple comparisons using Sidak’s multiple comparisons test. These differences are provided in Table 4. Two-way ANOVA was also used to look for mean differences in the cytokine and chemokine response to stroke in 3-month old versus 18-month old mice, and between a first and second stroke. This was followed by Sidak’s multiple comparisons test to look for significant differences in individual cytokines and chemokines between these experimental groups (Figs. 7 and 8).

## Results

### Histopathological staging of the human tissue

Fresh frozen brain tissue from acute and chronic stroke infarcts, and age-matched control brain tissue, was requested from the Brain and Body Donation Program at the Banner Sun Health Research Institute, the Human Brain and Spinal Fluid Resource Center at the University of California, Los Angeles, and the University of Miami Brain Endowment Bank. This resulted in the acquisition of tissue samples from 55 post-mortem brains; 33 samples came from patients with a clinical and neuropathological diagnosis of either acute or chronic stroke, and 23 samples came from age-matched control patients. The autolysis cut-off time for this study was 24 h, and the average autolysis time for each group ranged from 5.3 to 11.7 h (see Table 1 for patient information). Samples were dissected into two halves, one half was used for H&E staining and immunostaining, and the other half was used to generate lysates for multiplex immunoassays (Fig. 1a).

Due to a lack of clinical data to indicate the interval between stroke and death for many of the patients, and thus the age of the infarcts, samples were staged into three easily distinguishable categories that represent distinct phases of infarct resolution in the brain. Staging was performed by cross-referencing clinical data supplied by the tissue banks with H&E histopathology. The staging categories were (i) normal control tissue, (ii) acute lesions, (iii) lesions at the stage of liquefactive necrosis, and (iv) lesions at the stage of cystic encephalomalacia, the final stage of infarct resolution.

To be classified as normal control tissue, tissue had to appear homogenous and organized with typical-looking nuclei. A representative image of an H&E stain of normal control brain tissue is shown in Fig. 1b, panel (**i**). To be classified as a stroke lesion at the acute stage of infarction (i.e., hours old) samples met our criteria if we could confirm the presence of eosinophilic degenerating neurons, with an angular shape, vacuolation in the cytoplasm, and a shrunken darkly stained pyknotic nucleus [16]. A representative image of an H&E stain of a stroke lesion at the acute stage of infarction is shown in Fig. 1b, panel (**ii**). Samples were classified as at the stage of liquefactive necrosis (i.e. weeks to months old) if they were principally comprised of foamy macrophages, as shown in panel (**iii**) of Fig. 1b, and surrounded by an astroglial scar (not shown in the image). Finally, as seen in panel (**iv**) of Fig. 1b, lesions were classified as at the stage of cystic encephalomalacia (months to years old) if the infarct had been predominantly resorbed, leaving empty spaces and a dense network of glial processes and blood vessels.

### Controlling for autolysis

Autolysis causes protein degradation, but how rapidly this affects measurement of cytokine and chemokine concentrations in the brain during the first 24 h post-mortem is largely unknown [7, 24, 28]. Therefore, following histological staging, lysates prepared from the control tissue were used for multiplex immunoassay to evaluate whether there was a correlation between autolysis time and cytokine and chemokine concentrations in the cohort of control tissue. We performed this analysis on control tissue to avoid the confounding impact of stroke. A significant positive or negative correlation between autolysis time and cytokine and chemokine concentration would indicate that protein degradation is a confounding factor for the measurement of cytokine and chemokine concentrations in the brain by multiplex immunoassay in the first 24 h post-mortem. However, as shown in Table 3 there was no correlation between autolysis time and any cytokine and chemokine concentration in the brain. For this validation we included fractalkine, which unlike many of the other cytokines and chemokines, is highly expressed even at basal levels in healthy brain tissue [35].

**Table 3:** There is no correlation between autolysis time and cytokine and chemokine concentration in human control brain samples

Cytokine/chemokine	Pearson's r value	P value (two-tailed)	P value summary
GM-CSF	0.0870	0.7314	ns
Fractalkine	−0.0675	0.7901	ns
IFNγ	−0.2062	0.4116	ns
IL-10	0.2294	0.3597	ns
MIP-3α	0.1972	0.4328	ns
IL-12 (p70)	0.1332	0.5983	ns
IL-13	0.3367	0.1718	ns
IL-17A	0.0125	0.9608	ns
IL-1α	0.0285	0.9107	ns
IL-2	0.0435	0.8639	ns
IL-21	0.0321	0.8995	ns
IL-4	−0.0020	0.9939	ns
IL-23	−0.0045	0.9857	ns
IL-5	0.0172	0.9460	ns
IL-6	0.3862	0.1134	ns
IL-7	0.1697	0.5007	ns
IL-8	−0.1325	0.6001	ns
MIP-1α	0.0968	0.7025	ns
MIP-1β	−0.0552	0.8278	ns
TNFα	−0.1334	0.5976	ns
G-CSF	0.0057	0.9822	ns
IL-15	−0.1552	0.5386	ns
IL-1α	−0.1725	0.5080	ns
IL-9	0.0256	0.9198	ns
IP-10	−0.2842	0.2531	ns
MCP-1	0.1016	0.6882	ns
RANTES	0.1229	0.6269	ns

*ns* nonsignificant based on significance set at *p* < 0.05

### Cytokine and chemokine levels in human stroke infarcts at the stages of acute stroke, liquefactive necrosis, and cystic encephalomalacia

Lysates prepared from each sample were then used for multiplex immunoassays to determine what cytokines and chemokines are present in the human brain at the acute stage of stroke, as well as at the later stages of liquefactive necrosis and cystic encephalomalacia. In human acute infarcts, we noted increased levels of 19 out of 25 cytokines and chemokines compared to control tissue (Fig. 2a): GM-CSF, IFNγ, IL-1α, IL-1β, IL-2, IL-4, IL-6, IL-10, IL-12 (p70), IL-13, IL-15, IL-17, IP-10, IL-8, MCP-1, MIP-1α, MIP-1β, RANTES, and TNFα. If corrected for multiple comparisons, there were significantly higher levels of IL-1α, IL-2, IL-4, IL-6, IL-10, IL-12 (p70), IL-15, IL-17, IP-10, IL-8, MCP-1, MIP-1α, MIP-1β, RANTES, and TNFα.

The levels of most of these cytokines and chemokines were substantially decreased by 10–100 fold in the infarcts at the stage of liquefactive necrosis. Despite this resolution, several cytokines and chemokines remained elevated compared to control tissue: IL-6, IL-12 (p70), IL-8, and MCP-1 (Fig. 2b). If corrected for multiple comparisons, there were significantly higher levels of IP-10, MIP-1α, and MIP-1β.

Levels of several cytokines and chemokines were also higher than in controls in infarcts at the stage of cystic encephalomalacia: G-CSF, IFNγ, IL-6, IL-7, IL-10, IL-15, IP-10, IL-8, MCP-1, and RANTES (Fig. 2c). If corrected for multiple comparisons, there were significantly higher levels of G-CSF, IL-7, IL-10, IL-15, IP-10, and MCP-1. Surprisingly, levels of several cytokines and chemokines were decreased compared to control tissue: IL-1β, IL-2, IL-5 IL-12 (p70), IL-13, IL-17 and MIP-1β. If corrected for multiple comparisons there were significantly lower levels of IL-2 and IL-12 (p70).

These data demonstrate that there is substantial resolution of the inflammatory response to stroke between the acute time period and the stages of liquefactive necrosis and cystic encephalomalacia. However, they also suggest that many pro-inflammatory cytokines and chemokines (IL-6, RANTES, IL-12 (p70), MCP-1, MIP-1α, MIP-1β) [10, 38] remain chronically elevated at low levels within infarcts resolving by liquefactive necrosis, a process that proceeds for an unknown length of time in the human brain following stroke.

### Cytokine and chemokine levels in mouse infarcts at 24 h, 1 week, and 7 weeks following stroke

To reveal similarities and differences between mice and humans, we cross-matched the human multiplex immunoassay data with immunoassay data from mice. We used two popular inbred strains of mice, C57BL/6 and BALB/c mice, to control for mouse strain-related differences in the inflammatory response to stroke. After inducing strokes, mice were euthanized at three different time-points: 24 h following stroke to model acute stroke in humans, 1 week following stroke to model sub-acute stroke in humans, and 7 weeks following stroke to model infarcts at the stage of liquefactive necrosis in humans. We were unable to model the stage of cystic encephalomalacia because infarcts from mice euthanized as late as 14 weeks following stroke were still at the stage of liquefactive necrosis (data not shown).

As seen in Fig. 3a (left graph), there were increased levels of the following cytokines and chemokines in the lesion in C57BL/6 mice at 24 h post-stroke compared to the equivalent cortical location from control sham mice: G-CSF, GM-CSF, IL-5, IL-6, IL-12 (p70), IP-10, KC (homologue to IL-8 in humans), MCP-1, MIP-1α, MIP-1β, MIP-2A, RANTES, and TNFα. The level of IL-9 was decreased in C57BL/6 mice at 24 h post-stroke compared to sham mice. If corrected for multiple comparisons, there were significantly higher levels of MCP-1 and MIP-1β.

There was substantial overlap in the inflammatory response to acute stroke in BALB/c mice compared to C57BL/6 mice at 24 h post-stroke. In BALB/c mice there was an increase in G-CSF, GM-CSF, IFNγ, IL-1α, IL-1β, IL-2, IL-4, IL-5, IL-7, IL-9, IL-10, IL-12 (p40), IL-12 (p70), IL-13, IL-15, IL-17, IP-10, KC, MCP-1, MIP-1α, MIP-1β, MIP-2A, RANTES, and TNFα in the lesion compared to an equivalent area of cortex from sham controls (Fig. 3a, right graph). If corrected for multiple comparisons, there were significantly higher levels of G-CSF, GM-CSF, IL-1α, IL-5, IL-7, IL-12 (p40), IL-15, IP-10, KC, MCP-1, MIP-1α, MIP-1β, MIP-2A, and TNFα.

In C57BL/6 mice at 1 week post-stroke, despite substantial resolution, G-CSF, GM-CSF, IFNγ, IL-1α, IL-1β, IL-4, IL-5, IL-6, IL-10, IL-12 (p40), IL-12 (p70), IP-10, KC, MCP-1, MIP-1α, MIP-1β, MIP-2A, RANTES, and TNFα remained elevated in the infarct compared to the equivalent area of cortex from sham mice (Fig. 3b, left graph). If corrected for multiple comparisons, there were significantly higher levels of IP-10 and RANTES compared to sham mice. In BALB/c mice at 1 week post-stroke G-CSF, IP-10, KC, MCP-1, MIP-1α, and MIP-1β were increased, and levels of IL-1β, IL-10, and IL-13 were decreased compared to sham mice (Fig. 3b, right graph). If corrected for multiple comparisons, there were significantly higher levels of IP-10 and MIP-1β.

In C57BL/6 mice at 7 weeks post-stroke there were increased levels of G-CSF, GM-CSF, IFNγ, IL-1α, IL-5, IL-6, IL-12 (p40), IL-12 (p70), IP-10, KC, MCP-1, MIP-1α, MIP-1β, MIP-2A, RANTES, and TNFα within the infarct compared to the equivalent area of cortex from sham mice (Fig. 3c, left graph). If corrected for multiple comparisons, there were significantly higher levels of G-CSF, GM-CSF, IL-5, IL-6, IL-12 (p70), IP-10, KC, MCP-1, MIP-1α, MIP-1β, MIP-2A, RANTES, and TNFα compared to sham mice.

In BALB/c mice at 7 weeks post-stroke, levels of G-CSF, IL-2, IL-6, IL-17, IP-10, MCP-1, and RANTES were increased, and levels of GM-CSF, IFNγ, IL-10 were decreased compared to sham mice (Fig. 3c, right graph). If corrected for multiple comparisons there were significantly higher levels of IL-17 and MCP-1, and a significantly lower level of IFNγ.

As seen by comparing Figs. 2a and 3a, the cytokines and chemokines that were uniformly increased in the acute infarcts from C57BL/6 mice, BALB/c mice, and humans were GM-CSF, IL12 (p70), IP-10, KC/IL-8, MCP-1, MIP-1α, MIP-1β, RANTES, and TNFα. This shared expression pattern of cytokines and chemokines indicates that there is substantial overlap in the inflammatory response to stroke in acute infarcts between humans and these two mouse strains at 24 h post-stroke.

### Correlation of the acute inflammatory response to stroke in mice and humans

The preceding data was then analyzed to determine the degree of correlation between C57BL/6 mice and BALB/c mice to identify strain similarities and differences, and the degree of correlation between each mouse strain and humans to identify conserved cytokine and chemokine expressions.

We found a significant correlation in the acute cytokine and chemokine response to stroke between C57BL/6 and BALB/c mice at 24 h post-stroke (*R*^2^ = 0.9848, *p* < 0.0001; Fig. 4a), and at 1 week post-stroke (*R*^2^ = 0.8930, *p* < 0.0001; data not shown). These data indicate that with regard to the cytokines and chemokines measured in this study, inflammation during acute and sub-acute stroke is not significantly different in C57BL/6 and BALB/c mice.

However, there was a lack of correlation between C57BL/6 mice and humans at the acute stage of stroke (*R*^2^ = 0.0021; *p* = 0.8357, Fig. 4b, solid line), or between BALB/c mice and humans at the acute stage of stroke (*R*^2^ = 0.0039; *p* = 0.7782, Fig. 4c, solid line). When we performed a two-way ANOVA that was corrected for multiple comparisons, we found that G-CSF, IL-6, and KC/IL-8 were significantly different between C57BL/6 mice and humans, and G-CSF and IL-6 were significantly different between BALB/c mice and humans at this acute stage of stroke (Table 4).

**Table 4:** Cytokines and chemokines that are significantly different between strains and species

Comparison	Cytokines and chemokines that are significantly different by a two-way ANOVA corrected for multiple comparisons
Human acute vs C57BL/6 24 h stroke	G-CSF, IL-6, KC/IL-8
Human acute vs BALB/c 24 h stroke	G-CSF, IL-6
C57BL/6 vs BALB/c week 7 stroke	G-CSF, IL-17, IP-10, KC, MCP-1, MIP-1β, RANTES
Human liquefactive necrosis vs C57BL/6 week 7 stroke	G-CSF, MIP-1α
Human liquefactive necrosis vs BALB/c week 7 stroke	G-CSF

The acute cytokine and chemokine response to stroke in C57BL/6 mice still failed to show a significant correlation with humans when G-CSF, IL-6, and KC/IL-8 were excluded from the calculation of the R^2^ value (*R*^2^ = 0.0254, *p* = 0.5018; Fig. 4b, dotted line). However, the acute cytokine and chemokine response to stroke in BALB/c mice did correlate with humans when G-CSF and IL-6 were excluded from the R^2^ calculation (*R*^2^ = 0.1920, *p* = 0.0470; Fig. 4c, dotted line).

These data suggest that there may be a differential regulation of G-CSF and IL-6 between humans and these two mouse strains, and that there is a stronger conservation of the acute cytokine and chemokine response to stroke between BALB/c mice and humans as compared to C57BL/6 mice and humans.

### Correlation of the chronic inflammatory response to stroke in mice and humans

Along with overlap in the acute inflammatory response to stroke, there was also overlap in the inflammatory response to stroke in infarcts at the stage of liquefactive necrosis. IL-6 and MCP-1 levels were increased in areas of liquefactive necrosis in humans and both strains of mice (Figs. 2b and 3c).

With regard to correlation, we discovered a significant correlation in the cytokine and chemokine levels present in the infarcts dissected from C57BL/6 mice and BALB/c mice at 7 weeks following stroke (*R*^2^ = 0.8929, *p* < 0.0001; Fig. 4d). However, a two-way ANOVA corrected for multiple comparisons revealed that despite this correlation, there were still significant differences in the levels of G-CSF, IL-17, IP-10, KC, MCP-1, MIP-1β, and RANTES between these two strains (Table 4). These data indicate that despite a significant correlation, there is still a divergence in the chronic inflammatory response to stroke in C57BL/6 and BALB/c mice at the stage of liquefactive necrosis.

At the stage of liquefactive necrosis, the cytokine and chemokine response to stroke failed to correlate between C57BL/6 mice and humans (*R*^2^ = 0.0468, *p* = 0.3215, Fig. 4e, solid line). Two-way ANOVA corrected for multiple comparisons showed significant differences in the levels of G-CSF and MIP-1α between these two groups (Table 4). When G-CSF and MIP-1α were excluded from the calculation of the R^2^ value, the cytokine and chemokine response to stroke at the stage of liquefactive necrosis correlated between C57BL/6 mice and humans (*R*^2^ = 0.7818, *p* < 0.0001; Fig. 4e, dotted line). This suggests that although there is differential regulation of G-CSF and MIP-1α between C57BL/6 mice and humans at the stage of liquefactive necrosis following stroke, there is an otherwise strong correlation if these differences are taken into account.

At the stage of liquefactive necrosis, the cytokine and chemokine response to stroke also failed to correlate between BALB/c mice and humans (*R*^2^ = 0.1500, *p* = 0.0679; Fig. 4f, solid line). By a two-way ANOVA corrected for multiple comparisons, there was a significant difference in the levels of G-CSF between these two groups (Table 4). However, after G-CSF was excluded from the R^2^ calculation, the cytokine and chemokine response to stroke at the stage of liquefactive necrosis correlated between BALB/c mice and humans (*R*^2^ = 0.7265, *p* < 0.0001; Fig. 4f, dotted line). This suggests that similar to C57BL/6 mice, there is also differential regulation of G-CSF between BALB/c mice and humans at the stage of liquefactive necrosis, and if this difference is taken into account, then there is an otherwise strong correlation between BALB/c mice and humans.

### Composition of immune cell infiltrates in human and mouse infarcts at the stage of liquefactive necrosis

To further investigate heterogeneity in the inflammatory response to liquefactive necrosis, we next evaluated what immune cells are present in human and mouse infarcts during this stage of infarct resolution. Tissue underwent immunostaining for macrophages/microglia, T-lymphocytes, and B-lymphocytes. In both species, and each strain of mouse, CD68+ macrophages were uniformly present within the infarcts (Fig. 5a, c, d, and e). There were also T and B cells present within the infarcts in both strains of mice, and in most humans. However, in humans and each strain of mouse, there was a substantial difference in the extent of T-lymphocyte (CD4+ and CD8+ cells in humans and CD3+ cells in mice), and B-lymphocyte (CD20+ cells in humans and B220+ cells in mice) infiltration in the infarct (Fig. 5b and e). C57BL/6 mice had significantly greater numbers of T and B cells within the infarct than the BALB/c mice, and substantially more than in humans. In mice, this was due to a difference in delayed infiltration of T and B cells because there was no difference in the number of T and B cells present within lesions analyzed 1 week following stroke (Fig. 5e). The number of T and B cells present in the infarcts was more heterogeneous in the human patients than either inbred mouse strain, with some humans showing no evidence of B cells within the infarct (Fig. 5b and e).

Next, we compared the phenotype of the T-lymphocytes present in the mouse infarcts by flow cytometry with the goal of determining if there is heterogeneity not only in the magnitude of the adaptive immune response to stroke during infarct resolution, but also in the polarization of the adaptive immune response. We discovered that at 7 weeks post-stroke, the T cells present in the infarct in C57BL/6 mice had an increased polarization towards a Th1 phenotype when compared to BALB/c mice. This was evidenced by an increased prevalence of CD4+ cells in the infarct in C57BL/6 mice that were also positive for T-bet, a Th1 transcription factor, and IFN-γ, a key Th1 cytokine (Fig. 5f and j). In contrast, the T cells present in the infarct in BALB/c mice had an increased polarization towards a Th2 phenotype. This was evidenced by an increased prevalence of CD4+ cells in the infarct in BALB/c mice that were also positive for STAT6, a Th2 transcription factor, and IL-4, a key Th2 cytokine (Fig. 5g and j). The disparate polarization of T cells towards a Th1 or Th2 phenotype within the infarct in the different strains supports that inflammation may follow a different trajectory in different individuals during the stage of liquefactive necrosis.

In both strains, we found few CD4+ cells that were also IL-17+ within the infarcts (Fig. 5h and j), and no CD4+ cells that were also Foxp3+ (Fig. 5i and j). This indicates that in both strains, at the stage of liquefactive necrosis, there are few Th17 and regulatory T cells (Treg) present within the infarct.

### Correlation of the chronic inflammatory response to stroke in males and females

Biological sex is known to play a role in stroke incidence, prevalence, and severity [19], however no studies have investigated if the chronic inflammatory response to stroke is sexually dimorphic. Therefore, we investigated how well the chronic inflammatory response to stroke correlates in males and females in both C57BL/6 mice and in humans. We found a significant correlation in the cytokine and chemokine response to stroke between male and female C57BL/6 mice at 7 weeks post-stroke (*R*^2^ = 0.6199, *p* < 0.0001; Fig. 6a), and between male and female human infarcts at the stage of liquefactive necrosis (*R*^2^ = 0.1724, *p* < 0.0488; Fig. 6b). These data indicate that with regard to the cytokines and chemokines measured in this study, sexual dimorphism does not prevent the chronic inflammatory response to stroke in male and female C57BL/6 mice, and male and female humans, from correlating.

### Age moderately attenuates the chronic T cell response to stroke in 18-month old C57BL/6 mice

The human post-mortem tissue used in this study came from elderly individuals with an average age of 79.9 years and there is evidence to suggest that the inflammatory response to stroke is attenuated in aged mice [42]. To evaluate if age may explain why there was substantially less adaptive immune cell infiltration in the human stroke lesions at the stage of liquefactive necrosis than in the lesions from the 3-month old C57BL/6 mice, we determined whether the inflammatory response to stroke in C57BL/6 mice declines with age. To accomplish this goal, 18-month old C57BL/6 mice underwent DH stroke and were sacrificed 7 weeks later. As seen in Fig. 7a, the 18-month old C57BL/6 mice shared a similar cytokine and chemokine profile in the infarct compared to the 3-month old mice. There was no significant difference in the mean cytokine and chemokine levels by two-way ANOVA. However, there were differences in the levels of several cytokines and chemokines when corrected for multiple comparisons; KC was decreased, and RANTES was increased.

With regard to immune cell infiltration, there was no difference in B cell infiltration, but there was a significant reduction in T cell infiltration into the area of liquefactive necrosis in the aged mice. Nevertheless, even with this reduced infiltration the number of T cells within the infarct in the aged mice was significantly greater than the number of T cells present within the infarcts from the human samples (data not shown). Thus, although we detected a moderate attenuation of the inflammatory response in the aged mice with regard to T cell infiltration, it was not sufficient to explain why the inflammatory response to liquefactive necrosis is disparate in C57BL/6 mice and humans.

### Prior stroke exacerbates the inflammatory response to stroke in mice

Presently, little is known about the interaction of multiple sequential ischemic strokes on the inflammatory response to stroke in the human brain, despite the fact that 25 % of the approximately 800,000 strokes that occur in the United States every year are recurrent strokes [32]. There is substantial evidence from animal models that demonstrates that a non-injurious mild ischemic insult can confer transient preconditioning protection against a second insult [20, 45]. On the contrary, evidence also suggests that once a threshold of injury has been reached, a mild ischemic stroke followed by a second stroke can exacerbate brain damage [6]. Therefore, because many of the human samples we analyzed came from individuals that had suffered multiple strokes, we sought to determine (i) the impact of multiple strokes on the brain’s immune response, and (ii) whether the impact of multiple strokes differs in mouse strains.

C57BL/6 and BALB/c mice underwent DH or DMCAO stroke respectively, followed by an equivalent second stroke on the contralateral hemisphere 3 weeks later. After 24 h, 1 week, and 7 weeks following the second stroke, the second stroke lesions were dissected and multiplex immunoassays were performed.

As seen in Fig. 8a, in the C57BL/6 mice at 24 h following stroke, there was a significant difference in the mean cytokine and chemokine levels of the second stroke compared to the first stroke by two-way ANOVA (*p* = 0.0170). With correction for multiple comparisons, there was significantly more G-CSF and IL-6 present in the second infarct. In BALB/c mice (Fig. 8b), there was no significant difference in the overall cytokine and chemokine profile for one stroke versus two strokes at 24 h by two-way ANOVA (*p* = 0.3327).

At 1 week post-stroke in C57BL/6 mice, there was no significant difference in the overall cytokine and chemokine profile between the first and second stroke by two-way ANOVA (*p* = 0.8209) (Fig. 8c). Likewise, at 1 week post-stroke, there was no significant difference in the overall cytokine and chemokine profile by two-way ANOVA between the first and second stroke in BALB/c mice (Fig. 8d) (*p* = 0.6196).

However, at 7 weeks post-stroke in C57BL/6 mice, there was a significant difference in the overall cytokine and chemokine profile between the first and second stroke by two-way ANOVA (*p* < 0.0156; Fig. 8e). This difference in one versus two strokes is highlighted by a significant increase in the level of RANTES when corrected for multiple comparisons. Similarly, in BALB/c mice, there was also a significant difference in the overall cytokine and chemokine profile between the first and second stroke by two-way ANOVA (*p* < 0.0001; Fig. 8f). However, there was a significant increase in the levels of IL-4 and MIP-1α in this strain when corrected for multiple comparisons.

Together, these data indicate that although the acute and chronic inflammatory response to a second stroke is remarkably consistent, there does appear to be a mild but significant shift towards a more pro-inflammatory chronic response to a recurrent stroke, with the characteristics of the shift being strain dependent.

## Discussion

Despite decades of stroke research, the cytokine response to stroke in the healing human brain is largely uncharacterized. One of the reasons for the scarcity of this kind of published data is the challenge to obtain tissue from individuals that have died at multiple synchronized stages of infarct resolution following stroke, and died without a confounding neurological co-morbidity such as Alzheimer’s disease. Due to this limitation, we were only able to accurately stage and analyze tissue from 33 patients with a clinical and neuropathological diagnosis of either acute or chronic stroke. In light of this small cohort there are likely to be type I errors in the data analyzed without correction for multiple comparisons, and the data analyzed with correction for multiple comparisons may exclude legitimate differences. To enable future studies to differentiate signal from noise, and to compensate for this small cohort, we present data analyzed by both statistical approaches. We also performed a parallel analysis of the cytokine and chemokine response to stroke in the healing mouse brain. Overlap between the two species, in terms of which cytokines and chemokines are increased and decreased, are more likely to represent legitimate and translatable findings with regard to the inflammatory response to stroke in the human brain.

A small sample size is not the only limitation of this study. Autolysis is a significant challenge for multiplex immunoassay investigations of human brain due to the difficulty of obtaining post-mortem tissue rapidly. However, despite autolysis causing protein degradation, multiple studies have shown that many human brain proteins are relatively stable with respect to post-mortem intervals (PMI) of less than 24 h [7, 24, 28]. On the other hand, multiple studies have also shown that the abundance and integrity of proteins within the human brain can exhibit marked intercase variability due to such factors as whether an individual died of a prolonged illness with progressive hypoxia, or died suddenly of a cardiac arrhythmia [7, 24, 28]. These discrepancies make comparisons between groups difficult because there is likely to be large intra-group variability in these parameters. These parameters cannot be easily controlled for without conducting a rigorous prospective study. However, we found no correlation between autolysis time and cytokine and chemokine levels in the cohort of human control tissue. Although this finding does not definitively rule out the possibility that levels of cytokines and chemokines measured in this study were significantly altered by autolysis, it does provide additional assurance of the validity of the measurements. Furthermore, because autolysis time was kept to less than 5 min for the mice used in this study, performing a parallel analysis of the cytokine and chemokine response to stroke in the human and mouse brain also mitigates this limitation.

There are significant differences in the immune response to stroke in different mouse strains with regard to the frequency and distribution of different types of immune cells such B-lymphocytes, neutrophils, monocytes, and macrophages, and in the polarization of T cell responses [4]. To control for these strain differences, we used both C57BL/6 and BALB/c mice. These two strains are known to have significant differences in their immune response and susceptibility to autoimmune diseases. C57BL/6 mice represent a canonical Th1 mouse strain, while BALB/c mice represent a canonical Th2 mouse strain [49].

With regard to which cytokines and chemokines are increased in both humans and mice in acute stroke, we found that GM-CSF, IL12 (p70), IP-10, KC/IL-8, MCP-1, MIP-1α, MIP-1β, RANTES, and TNFα are significantly higher in both species, and in both strains of mice compared to non-stroked controls. The putative role and functional relevance of each of these cytokines and chemokines is summarized in Table 5.

**Table 5:** Putative role(s) of cytokines and chemokines that are increased in both human and mouse stroke

Cytokine/chemokine	Time-point	Putative role	Selected references
GM-CSF	Acute	Hematopoietic growth factor that plays a neuroprotective and angiogenic role in animal models of stroke.	[33, 47]
IL12 (p70)	Acute	Sub-unit of the pro-inflammatory cytokine IL-12. Produced by antigen presenting cells. Co-factor for the polarization of T cells towards Th1 cell-mediated immunity. Role in animal models of stroke unknown.	[50]
IP10	Acute	Pro-inflammatory chemokine for monocytes and Th1 cells. Role in animal models of stroke unknown.	[41]
KC/IL-8	Acute	KC and IL-8 are pro-inflammatory chemokine homologues that promote neutrophil recruitment to sites of tissue damage. Targeting neutrophils has mixed effects in animal models of stroke. Neutrophil blockade has so far failed to show therapeutic benefit at clinical trial.	[12]
MCP-1	Acute	Chemokine for monocytes, hematogenous macrophages, neutrophils, memory T-lymphocytes and natural killer cells. Increases infarct size in animal models of stroke. However, MCP-1/CCR2 signaling has also been shown to reduce hemorrhagic transformation in animal models of stroke.	[21, 46]
MIP-1α	Acute	Chemokine for macrophages, monocytes and neutrophils. Ligand for CCR5. CCR5 deficient mice have increased brain damage after ischemic stroke. However, mice lacking CCR5 in the periphery have reduced brain damage after ischemic stroke. The role of MIP-1α in mediating these mixed results is unknown.	[44]
MIP-1β	Acute	Chemokine for macrophages, monocytes and neutrophils. Also a ligand for CCR5. The role of MIP-1β in mediating the mixed results of targeting CCR5 signaling following stroke is unknown.	[11, 44]
RANTES	Acute	Chemokine for multiple leukocyte subsets, including T cells. Also a ligand for CCR5. Evidence from animal models suggests that RANTES is a primary mediator of cerebral inflammation, blood-brain barrier dysfunction, and tissue damage following stroke	[48]
TNFα	Acute	Pleiotropic pro-inflammatory cytokine that can activate NF-kB signaling, MAPK signaling, and induction of apoptosis in target cells. Both injurious and beneficial roles of TNFα have been demonstrated. Blockade of TNFα reduces infarct volume in animal models of stroke, however pretreating mice with TNFα can be neuroprotective.	[2, 34]
IL-6	Liquefactive Necrosis	Pro-inflammatory cytokine secreted by T cells and macrophages. An acute increase in IL-6 in the CNS is neuroprotective. A prolonged increase is detrimental. Role in chronic stroke infarcts unknown.	[14]
MCP-1	Liquefactive Necrosis	Chemokine for monocytes, hematogenous macrophages, neutrophils, memory T-lymphocytes and natural killer cells. Role in chronic stroke infarcts unknown. However, prolonged MCP-1 elevation correlates with poor infarct resolution after spinal cord injury.	[18]

We also found that there was a significant correlation between the global cytokine and chemokine profile to acute stroke in each mouse strain. However, there was a clear difference in the acute cytokine and chemokine response to stroke in humans and mice in terms of the regulation of G-CSF. G-CSF was robustly increased in both C57BL/6 and BALB/c mice in acute stroke, but was not increased in acute stroke in humans. G-CSF has repeatedly been shown to be neuroprotective in mouse models of stroke, but recently failed a human clinical trial [36, 39, 40]. The difference in the regulation of G-CSF in the two species in response to stroke may also mean that the neuroprotective role that G-CSF plays in mice may be less relevant for humans.

With regard to sub-acute stroke, we found that there is substantial resolution of the inflammatory response to stroke in mice between 24 h and 1 week after stroke. However, in the days following stroke the inflammatory response in the brains of C57BL/6 and BALB/c mice begins to diverge. These findings suggest that although the initial response to stroke is very conserved, the resolution of inflammation may follow a different path in different individuals. We did not look at the inflammatory response to sub-acute stroke in humans because we were unable to obtain sufficient tissue that met our staging criteria for this time-point. Once tissue becomes available, we will investigate the inflammatory response to sub-acute stroke in humans in a follow-up study.

With regard to chronic stroke, MCP-1 and IL-6 were significantly elevated in both mice and humans in infarcts at the stage of liquefactive necrosis, and CD68+ microglia/macrophages were the predominant cell type. In mice, this stage lasts for at least 14 weeks, and it is unknown how long this stage lasts in humans, although it may vary depending upon the size and location of the infarct. Based on our unpublished observations in mice, we hypothesize that it likely lasts for substantially longer than 14 weeks even for very small infarcts. Both MCP-1 and IL-6 are associated with pro-inflammatory macrophages, which we interpret to mean that in both humans and mice the macrophage response to stroke does not resolve towards a reparative tissue remodeling phenotype as efficiently as the macrophage response to damage in other tissues [15]. A similar chronic activation of macrophages has been observed in the spinal cord following injury [18]. The putative role and functional relevance of MCP-1 and IL-6 in infarcts at the stage of liquefactive necrosis is also summarized in Table 5.

In this study, we did not evaluate polymorphonuclear granulocyte infiltration in areas of liquefactive necrosis. Although Enzmann and colleagues have shown the presence of polymorphonuclear granulocytes within cerebral vessels in infarcted human stroke tissue at early time points, it remains to be determined whether granulocytes are still present at the stage of liquefactive necrosis, and if they have extravasated into the infarct at this stage [13].

The overall cytokine and chemokine profile in areas of liquefactive necrosis significantly correlated in each strain of mouse, and in humans and mice when G-CSF was excluded. However, there was heterogeneity in T- and B-lymphocyte infiltration into the lesion in each strain of mouse and in humans. In C57BL/6 mice, there were substantially more adaptive immune cells in the lesion than in either BALB/c mice or humans, and the T cells were more polarized towards a Th1 phenotype than in BALB/c mice. These data are in line with previous studies that have shown that C57BL/6 mice skew towards Th1 inflammatory responses, while BALB/c mice skew towards Th2 inflammatory responses [49].

The difference in the acute cytokine and chemokine profile between mice and humans, and the difference in the extent of chronic adaptive immune cell infiltration between C57BL/6 mice and humans, means that caution should be taken when using mice, and a single strain, to extrapolate the inflammatory response to stroke in humans. The strain heterogeneity also highlights the need to determine if there are some human stroke patients that have a more severe chronic inflammatory response to stroke than others. We recently demonstrated that targeting the B cell response to stroke in C57BL/6 mice can prevent the development of delayed cognitive deficits. Identifying human patients that have an equivalent B cell response to stroke may lead to significant recovery in this population by targeting B cells in the days following stroke [9].

The sizeable difference in the adaptive immune response to stroke in humans and C57BL/6 mice may be due to the advanced age of the human subjects compared to the young adult age of the C57BL/6 mice used in the first part of this study. Age has been reported to attenuate the inflammatory response to stroke in C57BL/6 mice [42]. To control for this possible confound, we compared the inflammatory response to stroke in both young adult and aged C57BL/6 mice. Although we did see significantly less KC present in the chronic infarcts of the aged mice, we detected significantly more RANTES, and the overall cytokine profile was unchanged by two-way ANOVA.

Surprisingly, in association with the increased amount of the T cell chemoattractant RANTES, there were fewer T cells within the infarcts of the aged mice compared to the young adult mice. This attenuated T cell response reinforces Sieber and colleagues’ findings that age attenuates the inflammatory response to stroke [42]. However, aged mice still had substantially more T cells within the lesion than humans, and so this attenuation is not considerable enough to explain the difference in the inflammatory response in areas of liquefactive necrosis in C57BL/6 mice and humans.

The human tissue used in this study came from both men and women. Our small sample size meant we were not sufficiently powered to look for individual sex differences in the inflammatory response to stroke in humans at each stage of infarct resolution. Therefore, we analyzed both sexes in combined groups. Nevertheless, to gather a broad sense of how much overlap there is in the chronic inflammatory response to stroke in men and women, we did calculate how well the sexes correlate with each other at this time-point. Our data revealed a significant correlation, which demonstrates that there is congruency in the chronic inflammatory response to stroke in men and women. The chronic inflammatory response to stroke also correlated in male and female C57BL/6 mice. Although these significant correlations provide an indication of how much overlap there is in each sex of each species, they do not imply that there are no sex differences in the inflammatory response to stroke in mice or humans.

Many of the human samples used in this study came from individuals that had suffered from multiple strokes. Consequently, it is possible that mice that have undergone multiple strokes could exhibit an inflammatory profile at the stage of liquefactive necrosis more equivalent to the human samples we analyzed. To test this likelihood we investigated the impact of multiple strokes on the cytokine and chemokine response to stroke in mice. We discovered that in mice, the inflammatory response to a recurrent stroke is very similar to the inflammatory response to a first stroke, although there is a mild exacerbation at 7 weeks post-stroke. This is in accordance with Clark and colleagues who demonstrated that a mild ischemic stroke followed by a second mild cerebral stroke exacerbates brain damage [6]. Nevertheless, the comorbidity of recurrent stroke did not make the inflammatory response to stroke in mice more similar to humans.

The findings presented in this study have therapeutic and diagnostic relevance. The therapeutic relevance is that extrapolating from the mouse data it appears that chronic infarcts persist at the stage of liquefactive necrosis for months following stroke. During this time there is a significant elevation of cytokines associated with proinflammatory marcophages (as summarized in Table 5). Therefore, there is a need to more fully characterize the phenotype of these macrophages in humans, as well as determine what factors they are producing, and for how long they produce them, so that they can be more effectively evaluated as a target for promoting recovery. The diagnostic relevance is that this study highlights the need for the development of live imaging techniques that can accurately differentiate between different stages of chronic infarct resolution. New methods need to be developed that not only distinguish liquefactive necrosis from cystic encephalomalacia, but also further subdivide these categories into intervening stages. This would greatly facilitate trials directed at mediating faster and more reparative infarct resolution in the brain following stroke.

## Conclusions

In conclusion, this study represents the first simultaneous characterization of the inflammatory response to stroke in the human and mouse brain at different stages of infarct resolution. We reveal important similarities and differences in the inflammatory response in these two species that will help in the selection and validation of targets for improving stroke recovery. Our findings reiterate the importance of taking strain into consideration when evaluating the inflammatory response to stroke, and demonstrate that not only are there important species and strain differences that should be taken into consideration when planning preclinical and clinical research, but that there are also differences in the inflammatory response depending on age and prior stroke history.

## References

[1] Barba R, Martinez-Espinosa S, Rodriguez-Garcia E, Pondal M, Vivancos J, Del Ser T (2000). Poststroke dementia : clinical features and risk factors. Stroke.

[2] Barone FC, Arvin B, White RF, Miller A, Webb CL, Willette RN, Lysko PG, Feuerstein GZ (1997). Tumor necrosis factor-alpha. A mediator of focal ischemic brain injury. Stroke.

[3] Beach TG, Adler CH, Sue LI, Serrano G, Shill HA, Walker DG, Lue L, Roher AE, Dugger BN, Maarouf C (2015). Arizona study of aging and neurodegenerative disorders and brain and body donation program. Neuropathology.

[4] Becker KJ. Strain-related differences in the immune response: relevance to human stroke. Transl Stroke Res. 2016. doi: 10.1007/s12975-016-0455-9. 10.1007/s12975-016-0455-9 PMC4929040 26860504

[5] Bejot Y, Aboa-Eboule C, Durier J, Rouaud O, Jacquin A, Ponavoy E, Richard D, Moreau T, Giroud M (2011). Prevalence of early dementia after first-ever stroke: a 24-year population-based study. Stroke.

[6] Clark D, Tuor UI, Thompson R, Institoris A, Kulynych A, Zhang X, Kinniburgh DW, Bari F, Busija DW, Barber PA (2012). Protection against recurrent stroke with resveratrol: endothelial protection. PLoS One.

[7] Crecelius A, Gotz A, Arzberger T, Frohlich T, Arnold GJ, Ferrer I, Kretzschmar HA (2008). Assessing quantitative post-mortem changes in the gray matter of the human frontal cortex proteome by 2-D DIGE. Proteomics.

[8] Doyle KP, Fathali N, Siddiqui MR, Buckwalter MS. Distal hypoxic stroke: a new mouse model of stroke with high throughput, low variability and a quantifiable functional deficit. J Neurosci Methods. 2012. doi: 10.1016/j.jneumeth.2012.03.003. 10.1016/j.jneumeth.2012.03.003 PMC3348433 22465679

[9] Doyle KP, Quach LN, Sole M, Axtell RC, Nguyen TV, Soler-Llavina GJ, Jurado S, Han J, Steinman L, Longo FM (2015). B-lymphocyte-mediated delayed cognitive impairment following stroke. J Neurosci.

[10] Duluc D, Delneste Y, Tan F, Moles MP, Grimaud L, Lenoir J, Preisser L, Anegon I, Catala L, Ifrah N (2007). Tumor-associated leukemia inhibitory factor and IL-6 skew monocyte differentiation into tumor-associated macrophage-like cells. Blood.

[11] Dziennis S, Mader S, Akiyoshi K, Ren X, Ayala P, Burrows GG, Vandenbark AA, Herson PS, Hurn PD, Offner HA (2011). Therapy with recombinant T-cell receptor ligand reduces infarct size and infiltrating inflammatory cells in brain after middle cerebral artery occlusion in mice. Metab Brain Dis.

[12] Easton AS (2013). Neutrophils and stroke - can neutrophils mitigate disease in the central nervous system?. Int Immunopharmacol.

[13] Enzmann G, Mysiorek C, Gorina R, Cheng YJ, Ghavampour S, Hannocks MJ, Prinz V, Dirnagl U, Endres M, Prinz M (2013). The neurovascular unit as a selective barrier to polymorphonuclear granulocyte (PMN) infiltration into the brain after ischemic injury. Acta Neuropathol.

[14] Erta M, Quintana A, Hidalgo J (2012). Interleukin-6, a major cytokine in the central nervous system. Int J Biol Sci.

[15] Ferrante CJ, Leibovich SJ (2012). Regulation of macrophage polarization and wound healing. Adv Wound Care.

[16] Garman RH (2011). Histology of the central nervous system. Toxicol Pathol.

[17] Gelderblom M, Leypoldt F, Steinbach K, Behrens D, Choe CU, Siler DA, Arumugam TV, Orthey E, Gerloff C, Tolosa E (2009). Temporal and spatial dynamics of cerebral immune cell accumulation in stroke. Stroke.

[18] Gensel JC, Zhang B (2015). Macrophage activation and its role in repair and pathology after spinal cord injury. Brain Res.

[19] Gibson CL (2013). Cerebral ischemic stroke: is gender important?. J Cereb Blood Flow Metab.

[20] Gidday JM (2006). Cerebral preconditioning and ischaemic tolerance. Nat Rev Neurosci.

[21] Gliem M, Mausberg AK, Lee JI, Simiantonakis I, van Rooijen N, Hartung HP, Jander S (2012). Macrophages prevent hemorrhagic infarct transformation in murine stroke models. Ann Neurol.

[22] Gupta A, Watkins A, Thomas P, Majer R, Habubi N, Morris G, Pansari K (2005). Coagulation and inflammatory markers in Alzheimer's and vascular dementia. Int J Clin Pract.

[23] Herson PS, Palmateer J, Hurn PD (2013). Biological sex and mechanisms of ischemic brain injury. Transl Stroke Res.

[24] Hynd MR, Lewohl JM, Scott HL, Dodd PR (2003). Biochemical and molecular studies using human autopsy brain tissue. J Neurochem.

[25] Iadecola C, Anrather J (2011). The immunology of stroke: from mechanisms to translation. Nat Med.

[26] Jin R, Yang G, Li G (2010). Inflammatory mechanisms in ischemic stroke: role of inflammatory cells. J Leukoc Biol.

[27] Levin EC, Acharya NK, Han M, Zavareh SB, Sedeyn JC, Venkataraman V, Nagele RG (2010). Brain-reactive autoantibodies are nearly ubiquitous in human sera and may be linked to pathology in the context of blood-brain barrier breakdown. Brain Res.

[28] Lewis DA (2002). The human brain revisited: opportunities and challenges in postmortem studies of psychiatric disorders. Neuropsychopharmacology.

[29] Leys D, Henon H, Mackowiak-Cordoliani MA, Pasquier F (2005). Poststroke dementia. Lancet Neurol.

[30] Margaritescu O, Mogoanta L, Pirici I, Pirici D, Cernea D, Margaritescu C (2009). Histopathological changes in acute ischemic stroke. Rom J Morphol Embryol.

[31] Mena H, Cadavid D, Rushing EJ (2004). Human cerebral infarct: a proposed histopathologic classification based on 137 cases. Acta Neuropathol.

[32] Mohan KM, Wolfe CD, Rudd AG, Heuschmann PU, Kolominsky-Rabas PL, Grieve AP (2011). Risk and cumulative risk of stroke recurrence: a systematic review and meta-analysis. Stroke.

[33] Navarro-Sobrino M, Rosell A, Penalba A, Ribo M, Alvarez-Sabin J, Fernandez-Cadenas I, Montaner J (2009). Role of endogenous granulocyte-macrophage colony stimulating factor following stroke and relationship to neurological outcome. Curr Neurovasc Res.

[34] Pan W, Kastin AJ (2007). Tumor necrosis factor and stroke: role of the blood-brain barrier. Prog Neurobiol.

[35] Paolicelli RC, Bisht K, Tremblay ME (2014). Fractalkine regulation of microglial physiology and consequences on the brain and behavior. Front Cell Neurosci.

[36] Ringelstein EB, Thijs V, Norrving B, Chamorro A, Aichner F, Grond M, Saver J, Laage R, Schneider A, Rathgeb F (2013). Granulocyte colony-stimulating factor in patients with acute ischemic stroke: results of the AX200 for Ischemic Stroke trial. Stroke.

[37] Ritzel RM, Capozzi LA, McCullough LD (2013). Sex, stroke, and inflammation: the potential for estrogen-mediated immunoprotection in stroke. Horm Behav.

[38] Roszer T (2015). Understanding the mysterious M2 macrophage through activation markers and effector mechanisms. Mediators Inflamm.

[39] Schabitz WR, Kruger C, Pitzer C, Weber D, Laage R, Gassler N, Aronowski J, Mier W, Kirsch F, Dittgen T (2008). A neuroprotective function for the hematopoietic protein granulocyte-macrophage colony stimulating factor (GM-CSF). J Cereb Blood Flow Metab.

[40] Schneider A, Kruger C, Steigleder T, Weber D, Pitzer C, Laage R, Aronowski J, Maurer MH, Gassler N, Mier W (2005). The hematopoietic factor G-CSF is a neuronal ligand that counteracts programmed cell death and drives neurogenesis. J Clin Invest.

[41] Seifert HA, Collier LA, Chapman CB, Benkovic SA, Willing AE, Pennypacker KR (2014). Pro-inflammatory interferon gamma signaling is directly associated with stroke induced neurodegeneration. J Neuroimmune Pharmacol.

[42] Sieber MW, Claus RA, Witte OW, Frahm C (2011). Attenuated inflammatory response in aged mice brains following stroke. PLoS One.

[43] Simone MJ, Tan ZS. The role of inflammation in the pathogenesis of delirium and dementia in older adults: a review. CNS Neurosci Ther. 2010. doi: 10.1111/j.1755-5949.2010.00173.x. 10.1111/j.1755-5949.2010.00173.x PMC6493838 20553303

[44] Sorce S, Bonnefont J, Julien S, Marq-Lin N, Rodriguez I, Dubois-Dauphin M, Krause KH (2010). Increased brain damage after ischaemic stroke in mice lacking the chemokine receptor CCR5. Br J Pharmacol.

[45] Stenzel-Poore MP, Stevens SL, Simon RP (2004). Genomics of preconditioning. Stroke.

[46] Strecker JK, Minnerup J, Gess B, Ringelstein EB, Schabitz WR, Schilling M (2011). Monocyte chemoattractant protein-1-deficiency impairs the expression of IL-6, IL-1beta and G-CSF after transient focal ischemia in mice. PLoS One.

[47] Sugiyama Y, Yagita Y, Oyama N, Terasaki Y, Omura-Matsuoka E, Sasaki T, Kitagawa K (2011). Granulocyte colony-stimulating factor enhances arteriogenesis and ameliorates cerebral damage in a mouse model of ischemic stroke. Stroke.

[48] Terao S, Yilmaz G, Stokes KY, Russell J, Ishikawa M, Kawase T, Granger DN (2008). Blood cell-derived RANTES mediates cerebral microvascular dysfunction, inflammation, and tissue injury after focal ischemia-reperfusion. Stroke.

[49] Watanabe H, Numata K, Ito T, Takagi K, Matsukawa A (2004). Innate immune response in Th1- and Th2-dominant mouse strains. Shock.

[50] Zaremba J, Losy J (2006). Interleukin-12 in acute ischemic stroke patients. Folia Neuropathol.

