# Erratum to: Multiplex immunoassay characterization and species comparison of inflammation in acute and non-acute ischemic infarcts in human and mouse brain tissue

**DOI:** 10.1186/s40478-016-0376-6

**Published:** 2016-09-26

**Authors:** Thuy-Vi V. Nguyen, Jennifer B. Frye, Jacob C. Zbesko, Kristina Stepanovic, Megan Hayes, Alex Urzua, Geidy Serrano, Thomas G. Beach, Kristian P. Doyle

**Affiliations:** 1 Department of Immunobiology, University of Arizona College of Medicine, Tucson, AZ USA; 2 Department of Neurology, University of Arizona College of Medicine, Tucson, AZ USA; 3 Arizona Center on Aging, University of Arizona College of Medicine, Tucson, AZ USA; 4 Banner Sun Health Research Institute, Sun City, AZ USA

## Erratum

*n.b. The errors and associated corrections described in this document concerning the original manuscript were accountable to the production department handling this manuscript, and thus are no fault of the authors of this paper. Additionally, the online manuscript has now been updated with these corrections accordingly.*

In the original publication of this article [1] the incorrect image was used for Fig. 7a. The previous image displayed significance stars over the wrong cytokines and as such, did not reflect the text in the methods, results or discussion. These should have been above the cytokines that were significant by 2way ANOVA corrected for multiple comparisons (KC and RANTES), as referenced in the rest of the manuscript.

**Fig. 7:**
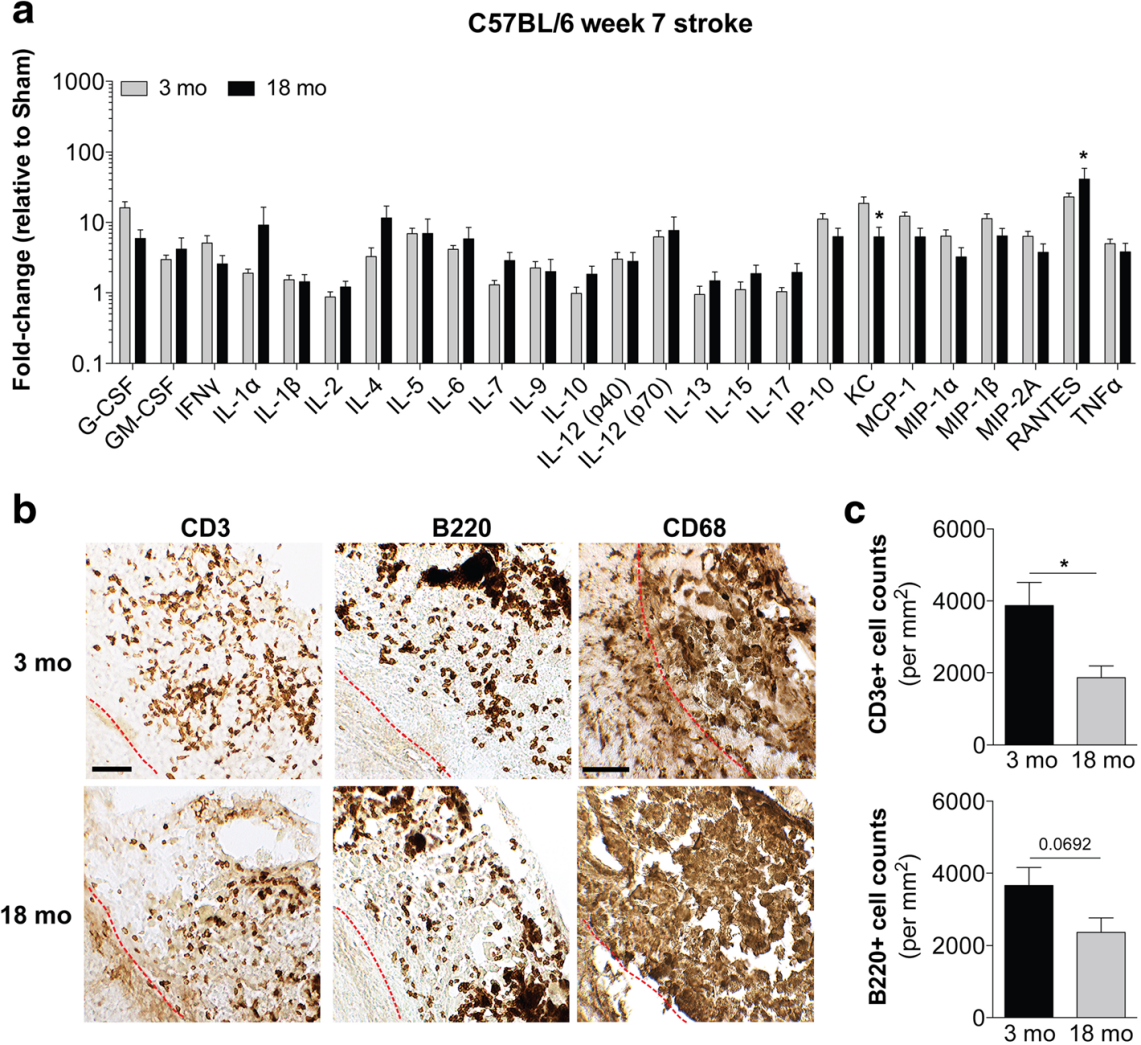
Impact of age on the chronic inflammatory response to stroke in C57BL/6 mice. **a** Comparison of the levels of 25 cytokines and chemokines in infarcts at the stage of liquefactive necrosis dissected from 3-month old and 18-month old C57BL/6 mice at 7 weeks post-stroke. Data are expressed as a fold-change relative to age matched sham control values. Data represent mean ± SEM. There is no significant difference in the overall cytokine and chemokine profile at the stage of liquefactive necrosis between 3-month old and 18-month old mice by two-way ANOVA. Cytokines and chemokines that are significantly different between the 3-month old and 18-month old mice corrected for multiple comparisons are denoted by an asterisk (*p < 0.05 versus 3-month old mice). **b** Representative images of CD3+ T-lymphocytes, B220+ B-lymphocytes, and CD68+ macrophages/microglia in the infarcts of 3-month old and 18-month old C57BL/6 mice at 7 weeks post-stroke. Scale bar, 50 μm. *Red dotted lines* indicate locations of the glial scar in each image. **c** Quantification of CD3+ T-lymphocyte and B220+ B-lymphocyte infiltration into the mouse infarcts

## References

[1] Nguyen et al. Multiplex immunoassay characterization and species comparison of inflammation in acute and non-acute ischemic infarcts in human and mouse brain tissue. Acta Neuropathologica Communications. 2016;4:100. doi:10.1186/s40478-016-0371-y. 10.1186/s40478-016-0371-y PMC5011964 27600707

